# The effect of the holiday season on body weight and composition in college students

**DOI:** 10.1186/1743-7075-3-44

**Published:** 2006-12-28

**Authors:** Holly R Hull, Casey N Hester, David A Fields

**Affiliations:** 1 Department of Health and Exercise Science, University of Oklahoma, Norman, OK, USA; 2 Department of Pediatrics, University of Oklahoma Health Sciences Center, Oklahoma City, OK, USA; 3 Children's Medical Research Institute's Metabolic Research Center, University of Oklahoma Health Sciences Center, Oklahoma City, OK, USA

## Abstract

**Background:**

With the rapid increase in obesity rates, determining critical periods for weight gain and the effects of changes in fat mass is imperative. The purpose of this study was to examine changes in body weight and composition over the holiday season (Thanksgiving through New Year's) in male and female college students.

**Methods:**

Subjects completed three visits: the first occurred within 2 weeks prior to Thanksgiving, the second occurred within 5 to 7 days following Thanksgiving, and the third occurred within 10 days following New Year's Day. A total of 82 healthy male and female college age subjects participated. Body composition by dual energy x-ray absorptiometry (DXA) was assessed at visits 1 and 3 while body weight was assessed at all three visits.

**Results:**

Average body weight remained relatively unchanged from pre-Thanksgiving to post-New Year's (71.3 ± 14 kg vs. 71.2 ± 15 kg; *P *= 0.71) and, in fact, a subset of normal weight subjects lost a significant amount of body weight. However, percent body fat (25.9 ± 9 %fat vs. 27.0 ± 9 %fat; *P *< 0.01) and fat mass (18.3 ± 8 kg and 19.1 ± 8 kg; *P *< 0.01) significantly increased from pre-Thanksgiving to post-New Year's while fat-free mass (48.7 ± 12 kg and 48.3 ± 11 kg; *P *= 0.08) was not significantly different than the post-New Year's. A significant positive relationship (*P *< 0.001) between the change in BMI and percent fat, total fat mass, total fat free mass, and trunk fat mass for the pre-Thanksgiving and post-New Year's visits were found. The same significant positive relationships (*P *< 0.001) were also observed between the change in body weight and percent fat, total fat mass, total fat free mass, and trunk fat mass.

**Conclusion:**

Despite the fact that body weight remained unchanged over the course of the holiday season, a significant increase in %body fat and fat mass was observed. With recent evidence showing marked morbidity and mortality to be associated with increased body fat (particularly abdominal adiposity), results from this study suggest body weight alone may underestimate the potentially deleterious effects of the holiday season.

## Background

Obesity has become a pervasive disease affecting all ages, socioeconomic classes, and ethnicities [1]. Current data indicates that obesity is responsible for approximately 300,000 deaths per year [2] with a direct impact of approximately 70 billion dollars on healthcare costs [3]. Though the obesity crisis is clearly visible, the exact mechanisms or time periods underlying the development of obesity are poorly understood. Given the recent and rapid rise in obesity, it is not likely due to changes in genetics or other biological causes, but rather changes in the environment which ultimately lead to a positive energy balance and weight gain [1].

Within this environmental model, certain phases of the year may represent critical time points for the development of obesity. Winter months in particular affect body weight via changes in food intake, mood and physical activity [4-9]. Notably, caloric intake during the fall is higher than in the spring, with peak caloric intake occurring during the month of November [4,10]. Concomitantly, physical activity levels have been shown to decline during cold weather months due to harsher temperatures and shorter amounts of daylight, further contributing to an overall increased risk for obesity during the fall and winter seasons [4,11].

A second critical time point for obesity development occurs during the college years [12], when healthy (or unhealthy) lifestyles may be adopted and carried on throughout adulthood. Unfortunately, recent studies indicate most college students are failing to develop healthy nutritional and physical activity habits [13]. In 1999, Mokdad et al. examined all age cohorts and found 18 to 29 year olds and those with some college education to be a group with the greatest increase in obesity [1]. Additionally, physical activity levels decline as transitions are made from adolescence into adulthood [14,15].

It is commonly reported by the media that 5 pounds of body weight is gained during the holidays, but little research using sophisticated methods to assess changes in body weight and fat mass distribution has been done to validate this claim [5-7,9,16]. Specifically, no study has assessed changes in fat mass and fat-free mass using dual energy X-ray absorptiometry (DXA) over the holiday season in college students. The purpose of this study was to examine the effect of the holiday season (i.e. Thanksgiving through New Year's) on body weight and body composition in college students.

## Research methods and procedures

### Subjects

Following approval by the University of Oklahoma-Norman Campus Institutional Review Board, 100 male and female students enrolled at the University of Oklahoma-Norman campus completed the first visit. The ages of students ranged from 18–40 years (23.0 ± 4.7 yrs) and represented a wide rang in class standing (i.e. freshman through graduate students). Subject recruitment occurred via mass email and announcements in college courses by instructors.

### Protocol

Data were collected at 3 time points throughout the holiday season, with the first visit taking place the week prior to the Thanksgiving holiday (Nov 14^th ^– 22^nd^) and the last visit occurring after the New Year holiday (Jan 9^th ^– Jan 21^st^). Subjects were brought back for a second visit 5–7 days following Thanksgiving (Nov 28^th ^– Dec 2^nd^). Visits 1 and 3 were identical, with body weight measured to the nearest 0.1 kg using a Detecto Manual Physician scale with subjects wearing light clothing (i.e. no sweaters, jackets, or belts) and no shoes. Height was measured to the nearest 0.1 of a centimeter using a stadiometer (Accu-Hite Wall Stadiometer, Seca Corp., Hanover, MD). Body composition and distribution were assessed using DXA. During visit 2, only body weight was obtained. Written informed consent was obtained from each subject prior to testing.

### Dual Energy X-ray absorptiometry

DXA was used to assess percent body fat, fat mass, and fat-free mass while all scans were performed and analyzed by the same trained technician (HH) using a Lunar DPX-IQ software version 4.7 b. The DXA was calibrated each day prior to the start of testing using a known calibration block. Subjects arrived at the laboratory after fasting (i.e. six hours) and refraining from exercise (i.e. twenty-four hours). All metal was removed and height and weight were measured before subjects were scanned. The subject was placed on the scanning table within the scan box and centered on the scan table. Anterior posterior thickness was measured at the midsection to determine the appropriate scan speed.

### Data analysis

Statistical analysis was performed using Statistical Package for Social Sciences version 11.5 (SPSS). The means and the standard deviations of body weight, body composition and distribution variables for pre-Thanksgiving, post-Thanksgiving and post-New Year's were calculated. Paired t-tests were used to analyze body weight and composition differences between visits 1 (pre-Thanksgiving) and 3 (post-New Year's). Pearson's correlations were used to assess relationships between changes in body weight and body mass index (BMI) with percent body fat, total fat mass, total fat-free mass, and trunk fat mass. Data are reported as mean ± standard deviation (SD) and statistical significance was set at *P *≤ 0.05.

## Results

Of 100 enrolled subjects, 82 completed all 3 visits and data presented is from these subjects. Baseline characteristics of completers and non-completers were compared and no differences between the groups were found. Additionally, no gender differences were found and analysis was performed on the entire group. Descriptive characteristics of the study sample are presented in tables 1 and 2.

**Table 1:** Baseline clinical/demographic characteristics of study completers (N = 82).

	**Count **(% of total)
Gender	

Male	37 (45%)
Female	45 (55%)

Ethnicity	

Caucasian	61 (73%)
African American	6 (7%)
Asian	3 (4%)
Hispanic	10 (12%)
Native American	4 (4%)

Class Standing	

Freshman	21 (25%)
Sophomore	7 (8%)
Junior	9 (11%)
Senior	20 (26%)
Graduate	25 (30%)

BMI (kg/m^2^)	

Normal (>24.9 kg/m^2^)	54 (66%)
Overweight/Obese (>25 kg/m^2^)	28 (34%)

**Table 2:** Subject characteristics at the baseline visit (i.e. before Thanksgiving) for all study completers (N = 82).

	Means ± SD
Age	23.0 ± 5
Height (cm)	172.3 ± 9
Weight (kg)	71.3 ± 14
BMI (kg/m^2^)	23.9 ± 4
Waist Circumference (cm)	79.0 ± 10
Hip Circumference (cm)	99.6 ± 9
Waist/Hip Ratio	0.79 ± 1

**Figure 1:**
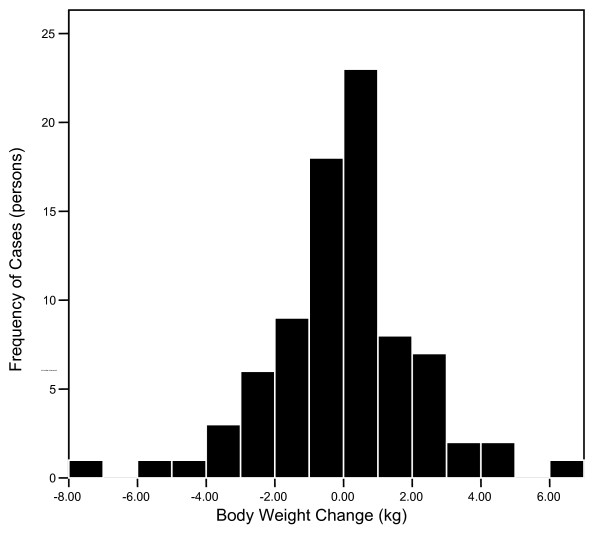
Distribution of change in weight of subjects completing the study pre-Thanksgiving to post-New Year (N = 82).

The distribution of weight changes between pre-Thanksgiving and post-New Year's for all subjects are shown in Figure 1. In the 31 subjects that gained weight, 12 of the subjects (15%) gained 2.0 kg or more over the course of the holiday season, 32 (39%) subjects actually lost body weight, and 19 (23%) were deemed weight stable (i.e. a change of ± 0.5 kg) from pre-Thanksgiving to post-New Year's. Thus, on average, body weight did not significantly increase from pre-Thanksgiving (71.3 ± 14 kg) to post-New Year's (71.2 ± 15 kg; *P *= 0.71).

**Figure 2:**
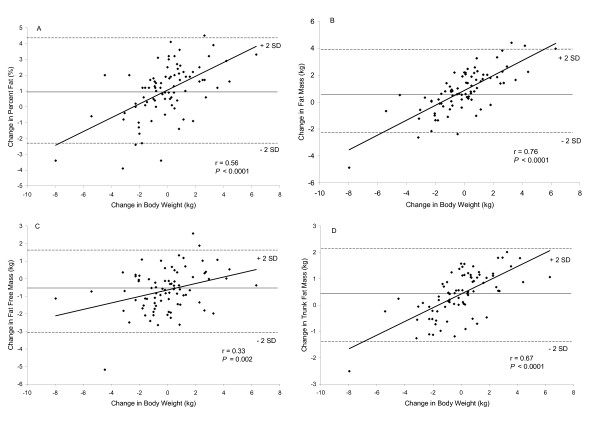
Relationship between change in body weight and percent fat, total fat mass, total fat free mass, and trunk fat mass (N = 82). The middle solid line represents the mean change of the x-axis variable and the upper and lower dashed line represents +2 standard deviations (SD) and -2 standard deviations (SD), respectively.

**Figure 3:**
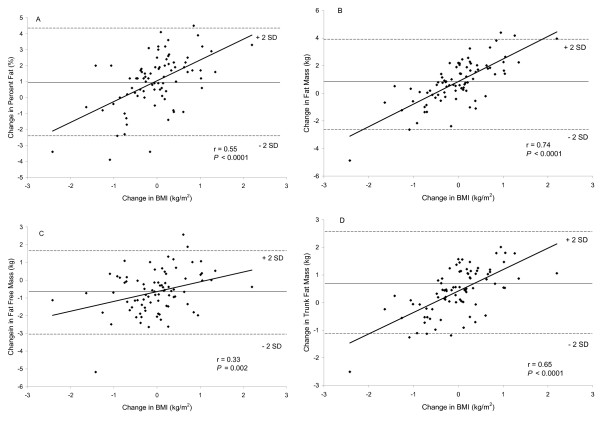
Relationship between the change in BMI and percent fat, total fat mass, total fat free mass, and trunk fat mass (N = 82). The middle solid line represents the mean change of the x-axis variable and the upper and lower dashed line represents +2 standard deviations (SD) and -2 standard deviations (SD), respectively.

Figure 2 depicts the relationships between the changes in body weight in relation to the change in percent fat, fat mass, fat free mass, and trunk fat mass. Positive significant relationships (*P *< 0.001) were found between changes in body weight and percent fat, total fat mass, total fat free mass, and trunk fat mass (panels A, B, C, and D). In those subjects judged to be weight-stable, 17 (89%) increased fat mass and 3 (16%) had an increased fat-free mass. Similar significant relationships (*P *< 0.001) were found between changes in BMI and percent fat, total fat mass, total fat free mass, and trunk fat mass (Figure 3 panels A, B, C, and D).

On average, a significant (*P *< 0.01) increase in percent body fat and total fat mass was observed between pre-Thanksgiving and post New Year's visits, while total fat-free mass remained unchanged (Table 3).

**Table 3:** Body weight and body distribution changes for all subjects (N = 82).

	Pre-Thanksgiving	Post-New Year
Body Weight (kg)	71.3 ± 14	71.2 ± 15
Body Fat (%)	25.9 ± 9	27.0 ± 9^†^
Total Fat Mass (kg)	18.3 ± 8	19.1 ± 8
Arm Fat Mass (kg)	1.7 ± 1	1.7 ± 1
Leg Fat Mass (kg)	7.2 ± 3	7.5 ± 3^†^
Trunk Fat Mass (kg)	8.3 ± 4	8.7 ± 4^†^
Total Fat-Free Mass (kg)	48.7 ± 12	48.3 ± 11
Arm Fat-Free Mass (kg)	6.0 ± 2	5.9 ± 2^†^
Leg Fat-Free Mass (kg)	17.1 ± 4	16.7 ± 4^†^
Trunk Fat-Free Mass (kg)	22.7 ± 5	22.6 ± 5

Means ± standard deviations

^†^Significantly different from Pre-Thanksgiving (*P *< 0.01)

In an attempt to gain a clearer picture of the impact of the holiday season on fat deposition, regional depots (i.e. arm, leg, and trunk) fat mass and fat free mass were investigated. Significant (*P *< 0.01) increases in trunk and leg fat mass were observed between the pre-Thanksgiving and post New-Year's visits, with a significant (*P *< 0.01) decrease observed in both leg and arm fat free mass (Table 3). No significant change in trunk fat free mass or arm fat mass was observed (Table 3).

To more clearly delineate the relationship between holiday weight gain and body weight status, subjects were divided into one of two groups, either normal body weight, defined as a BMI < 24.9 kg/m^2 ^(N = 54) or overweight/obese, defined as BMI ≥ 25 kg/m^2 ^(N = 28) (Table 4). The normal weight group lost a significant amount of body weight between the pre-Thanksgiving and post New Year's visits (*P *< 0.05) and had the largest number of individuals (9) who lost weight 2.0 kg or more, while the overweight/obese group had a greater number of subjects (8), who had gained more than 2.0 kg of body weight, although, on average, this was not significant (*P *= 0.14) Table 4.

**Table 4:** Body weight and body distribution changes by BMI classification.

	Normal (>24.9 kg/m^2^) (N = 54)		Overweight/Obese (>25 kg/m^2^) (N = 28)	
	Pre-Thanksgiving	Post-New Year	Pre-Thanksgiving	Post-New Year

Body Weight (kg)	64.1.7 ± 9	63.6 ± 9*	85.1 ± 12	85.9 ± 11
Body Fat (%)	23.5 ± 8	24.4 ± 8^†^	30.7 ± 9	31.9 ± 9*
Total Fat Mass (kg)	14.7 ± 5	15.2 ± 5^†^	25.5 ± 7	26.8 ± 7*
Arm Fat Mass (kg)	1.3 ± 1	1.3 ± 1	2.5 ± 1	1.8 ± 1
Leg Fat Mass (kg)	5.9 ± 2	6.1 ± 2*	9.7 ± 3	10.3 ± 3^†^
Trunk Fat Mass (kg)	6.4 ± 2	6.7 ± 2*	11.9 ± 3	12.5 ± 3^†^
Total Fat Free Mass (kg)	45.4 ± 10	44.9 ± 9^†^	55.4 ± 12	54.8 ± 12
Arm Fat Free Mass (kg)	5.5 ± 2	5.4 ± 2^†^	7.0 ± 2	6.8 ± 2*
Leg Fat Free Mass (kg)	16.0 ± 3	15.5 ± 3^†^	19.1 ± 4	18.9 ± 4
Trunk Fat Free Mass (kg)	21.0 ± 4	20.9 ± 4	25.9 ± 6	25.7 ± 6

Means ± standard deviations

*Significantly different from Pre-Thanksgiving (*P *< 0.05)

^†^Significantly different from Pre-Thanksgiving (*P *< 0.01)

Both the normal and overweight/obese groups gained a significant amount of body fat (*P *< 0.05) between the pre-Thanksgiving and New Year's visits (Table 4). A significant decrease in total fat-free mass in the normal weight group (*P *< 0.05) was observed, though the trend was the same for the overweight/obese group but it did not reach significance (*P *= 0.06) (Table 4). A summary of regional (i.e. arms, leg, and trunk) body composition for both the normal and overweight/obese groups are presented in Table 4. The general trend observed in both groups was for a significant increase in leg and trunk fat and a decrease in arm and leg fat free mass (Table 4).

## Discussion

This study sought to better understand the effect of the holiday season on body weight and composition in a group of college students. This is the first study that we are aware of utilizing a sophisticated method (i.e. DXA) to assess changes in body composition during the holiday season. Surprisingly, results from this study did not support the commonly held belief of significant weight gain over the holidays though 15% of our sample did gain greater than 2.0 kg of body weight.

Interestingly, this study showed no significant increase in body weight, yet total fat mass increased for the entire holiday season, which is in contrast to other studies examining the impact of the holiday season on body weight. Reid and Hackett examined the effect of Christmas on body weight and found a non-significant increase in body weight of 0.93 kg [9]. Possible limitations of the Reid study included the enrollment of only 26 subjects, with five subjects reported being ill. Yanovski et al. measured body weight in 195 adults during four time points ranging from pre-holiday (in late September or early October), to post-holiday (in January, February or March) [5]. Subjects were weighed again the following September. An increase in body weight of 0.37 kg (*P *< 0.001) was found during the holidays [5].

Results for weight gain specifically related to Thanksgiving in this cohort showed a significant (*P *< 0.05) increase of 0.5 kg of body weight [16]. When stratified by BMI, the overweight/obese group gained 1.0 kg of body weight (*P *< 0.05) whereas the normal BMI group gained a non-significant 0.2 kg of weight [16]. However, when subjects returned for follow-up after New Year's, body weight had returned to pre-holiday weight values. Though preliminary, these results suggest that subjects are attempting to maintain a "preset weight," as suggested by body weight returning to baseline values after the six week holiday period. Conversely, even though body weight returned to pre-holiday values, the percentage of body fat increased irrespective of BMI.

Although the holiday season lasts approximately six weeks, in that short period of time changes in total and regional fat mass and fat-free mass were observed. Positive associations were found between changes in body weight and fat mass, percent fat and trunk fat mass. Thus, as body weight increased, increases were seen in those compartments as well. A positive association, though not as strong, was found between change in body weight and fat free mass indicating some of the weight gained represented increases in muscle mass.

Perhaps most alarming is the significant increase in trunk fat mass found in both the normal (0.3 kg) and overweight/obese (0.6 kg) BMI groups. This is particularly worrisome given that excessive accumulation of trunk fat is related to a host of co-morbid conditions such as cardiovascular disease, type 2 diabetes, and early mortality [17-19]. Taken together, this study demonstrates that although body weight did not change, the impact of the holiday season played a crucial and deleterious effect on the shift of central body fat. This is provocative because most individuals judge overall health based on their body weight or BMI. In normal BMI subjects, weight returned to below baseline values at the end of the study even though percent body fat increased. Consequently, these subjects would have considered themselves as returning to their pre-holiday health status, when in fact, as a result of increasing total percent body fat (specifically trunk fat mass), they had actually increased their risk for the development of future disease.

In conclusion, on average no change was observed in body weight over the holiday season, although a rise in fat mass coupled with a decrease in fat-free mass resulted in a significant increase in percent body fat, specifically trunk fat mass. These findings were seen irrespective of BMI category. Though preliminary, this study shows that the holiday season may indeed have a deleterious impact on body composition that is not evident with the common bathroom scale. These findings raise the question: do the scales tell the whole truth?

## Competing interests

The author(s) declare that they have no competing interests.

## Authors' contributions

HH and DF conceived the study and wrote the manuscript while HH collected the data. CH provided critical review and revisions to the manuscript.
